# Autism and Dementia: A Summative Report from the 2nd International Summit on Intellectual Disabilities and Dementia

**DOI:** 10.1007/s10803-025-06843-7

**Published:** 2025-05-06

**Authors:** M. P. Janicki, P. McCallion, N. Jokinen, F. K. Larsen, D. Mughal, V. Palanisamy, F. Santos, K. Service, A. Shih, S. Shooshtari, A. Thakur, G. Tiziano, K. Watchman

**Affiliations:** 1 Institute of Disability and Human Development, University of Illinois Chicago, Chicago, USA; 2 School of Social Work and ABA Centers of America Autism Laboratory, Temple University, Philadelphia, PA USA; 3 School of Social Work, University of Northern British Columbia, Prince George, BC Canada; 4 Norwegian National Centre for Ageing and Health, Vestfold Hospital Trust, Tønsberg, Norway; 5 School of Public Health and Sciences, Gannon University, Erie, PA USA; 6 DDMHS/Fraser Health Authority, Burnaby, BC Canada; 7 School of Psychology, University College Dublin, Dublin, Ireland; 8 Massachusetts Department of Developmental Services, Northampton, MA USA; 9 Autism Speaks, Chicago, IL USA; 10 Department of Community Health Sciences, University of Manitoba, Winnipeg, MB Canada; 11 University of Toronto, Toronto, ON Canada; 12 Lega Del Filo D’Oro, Osimo, Italy; 13 Faculty of Health Sciences and Sport, University of Stirling, Stirling, Scotland; 14 PO Box 862, Rockport, ME 04856 USA

**Keywords:** Aging, Autism, ASD, Dementia, Neuropathologies, Risk factors

## Abstract

This article synthesizes findings, from the Autism/Dementia Work Group of the 2nd International Summit on Intellectual Disabilities and Dementia, on the nature of autism/autism spectrum disorder and later-age neuropathologies, particularly dementia. The convened group of experts explored genetic, neurobiological, and environmental risk factors that may affect the lifespan and lived experiences of older adults with autism. A review of current literature indicates a lack of comprehensive information on the demographics and factors associated with aging in autistic adults. However, our understanding of autism is evolving, challenging traditional views of it as a static, inherited neurodevelopmental disorder. The relationship between autism and other neurodevelopmental conditions—such as Down syndrome, fragile X syndrome, and tuberous sclerosis complex—reflects the complex genetic landscape of neurodevelopmental disorders. These genetic and familial factors may contribute to progressive health challenges and cognitive decline in later life. Key findings reveal a complex link between autism and dementia, despite limited research on this relationship, particularly among older adults. The overall prevalence of dementia in this population appears to be influenced by co-occurring intellectual disabilities, particularly Down syndrome. While the association between autism and specific types of dementia is still not well understood, the reviewed evidence suggests a notable connection with frontotemporal dementia, although causality has not been established. Exploration of biomarkers may offer further insights. Currently, the relationship between autism, cognitive health, and cognitive decline in older adults remains a complex and underexplored area of research.

## Introduction

The 2nd International Summit on Intellectual Disability and Dementia, held in Toronto, Canada, on October 24–25, 2023, aimed to explore the relationship between dementia and certain neurodevelopmental conditions, including autism spectrum disorder. Key topics include whether autism increases the risk for specific forms of dementia, how dementia manifests in autistic adults compared to the general population, and whether its presentation differs from that in other neurodevelopmental conditions.

In this summative article we present a synthesis of key issues identified and noted upon relative to autism and dementia within the Summit and derived from the Summit’s report, *Autism, Aging, and Dementia: A Consensus Report of the Autism/Dementia Work Group of the 2nd International Summit on Intellectual Disabilities and Dementia*, released by the Intellectual Disabilities/Dementia Summit Secretariat (International Summit Autism/Dementia Work Group, 2024). The full 104-page report covers the comprehensive review undertaken by the Autism/Dementia Work Group (A/DWG) which addressed the following areas: (a) key findings from previous research; (b) links between autism, intellectual disability (ID), and dementia; (c) contributors to an increased risk of dementia in individuals with autism and ID; (d) challenges in diagnosing dementia in older autistic adults; (e) functional non-pharmacological interventions and supports for those adults post-diagnosis and their care partners, and (f) a research agenda.

To effectively present the main findings on tasks a, b, and c, this article highlights key insights from existing literature on the etiology, comorbidities, and risk factors associated with dementia and autism. For detailed reviews and analyses, please refer to the full report from the International Summit Autism/Dementia Work Group (2024). Forthcoming articles will provide extracts of findings on the challenges of diagnosing dementia in older autistic adults [task d] and factors associated with non-pharmacological interventions and supports [task e] (see A/DWG-A, and A/DWG-B, in development).,^1^,^2^

The A/DWG’s work highlights how advancements in research methodologies and a more diverse range of study participants have significantly expanded our understanding of autistic adults. Earlier research often relied on narrow definitions of autism, primarily focusing on childhood within a psychiatric framework. Today, autism is increasingly studied in relation to other neurodevelopmental conditions, reflecting the complex genetic landscape of neurodevelopmental disorders. Genetic research, including data from the Simons Foundation Autism Research Initiative (SFARI) database, has identified over 1000 genes associated with autism, underscoring its broad and heterogeneous genetic basis (Iannuccelli et al., 2024). Contemporary studies now capture the full spectrum of autism across the lifespan, incorporating longitudinal designs and examining autistic adults beyond those with co-occurring intellectual disabilities (ID). This evolving research has deepened our understanding of how autistic traits change over time and intersect with other forms of neurodivergence. Additionally, there is growing recognition of the lived experiences of autistic adults, along with the various factors influencing their development, maturation, and aging.

In our work, we followed the established framework for defining autism as outlined by clinical standards organizations ([DSM-5] American Psychiatric Association, 2022; National Institute for Health Care and Excellence, 2021). We also acknowledged variations in preferred terminology, including terms like autistic spectrum condition, autistic spectrum difference, and neurodiversity (e.g. Canadian Academy of Health Sciences, 2022; Guideline Development Group, 2012—Pilling et al., 2012). Our analysis included studies that addressed both autism and autism spectrum disorder (ASD), and we chose to use identity-first language, such as"autistic adults"(Canadian Academy of Health Sciences, 2022; Edelson et al., 2021).

The contributions of prior work groups (e.g. Autism Canada, Autism Research Institute, and Pacific Autism Family Network Edelson et al., 2021; Happé & Charlton, 2012; Janicki et al., 2008; Mason et al., 2022; Mukaetova-Ladinska et al., 2012; Perkins & Berkman, 2012; Piven & Rabins, 2011; Roestorf et al., 2019) were considered. While previous studies and reports have laid some groundwork for exploring emergent neuropathologies, such as dementia in older adults with autism, they have not yet provided a definitive understanding of divergent aging in autism. There is broad agreement that the challenges in studying the intersection of autism and aging are considerable, due to limited case identification, the underrepresentation of older autistic adults in research, and the limited focus on aging within the autism research community. Additionally, the health status and expected trajectories of older autistic individuals remain largely unexplored, with little comprehensive insight into their phenomenology, associated features, secondary conditions, neurobiological changes, and specific medical, psychiatric, and social aspects. Research continues to focus primarily on children and young adults, leaving a significant gap in our understanding of aging, as well as the potential risk factors and biomarkers for late-onset mild neurocognitive disorders and dementia.

## Methodology

Summit participants included a diverse group of international researchers, academics, advocates, care providers, and government officials, all concerned with the limited understanding of dementia among adults with intellectual and developmental disabilities, including autism. Those with a focus on autism collaborated in small groups to create background documents, conducting thorough searches of both published and grey literature prior to the Summit. These documents served as a foundation for the Summit’s discussions. After the Summit, members of the A/DWG refined various drafts of the report. Near the end of the process, drafts were shared with all participants to gather additional insights and achieve consensus on the findings and recommendations. The final drafts were then reviewed by external content experts, who provided further input. Before publication, the report was vetted by the ID/Dementia Summit Secretariat and approved for web posting and distribution. Copies of the executive summary were also prepared for translation. Full details on the process and outcomes are available in the complete report (International Summit Autism/Dementia Work Group, 2024).

## Considerations

### Demographics

The available adult autism data primarily consists of prevalence studies on cognitive decline, with most aging estimates being extrapolations of epidemiological studies focused on early or school-age populations. Current information indicates that autism is present in about 2% of adults (Dietz et al., 2020), with an approximate 4:1 male-to-female ratio (Baron-Cohen et al., 2011; Zeidan et al., 2022). This prevalence does not significantly vary by race or ethnicity.

As autistic individuals transition into old age, the lifetime experience of autism may contribute to subtle and potentially early symptoms of mild neurocognitive disorders and dementia (Krantz et al., 2023). However, existing literature often fails to distinguish among factors derived from aging, autism, and comorbidities such as ID, which could influence the risk of cognitive decline in specific groups (International Summit Autism/Dementia Work Group, 2024). These groups may have also been overlooked in epidemiological studies of ID cohorts.

The demographic distribution of autistic individuals resembles a pyramid, with most people in the first three decades of life and progressively fewer in middle and older age (International Summit Autism/Dementia Work Group, 2024). This apparent “decline is likely due to several factors, including lower diagnostic rates and stricter criteria used in previous decades, which now affect cross-sectional demographic studies (Hayes et al., 2018). Other contributing factors include symptom moderation over time, earlier mortality, limited clinical contact, diagnostic uncertainties, increased societal integration, and challenges in identifying autism in older populations (Gerhardt & Lainer, 2011; Krantz et al., 2023; Shaw et al., 2023).

### Nutrition, Microbiota, and Gut Health and Autism

Our evolving understanding of autism is challenging the traditional view of it as a static, inherited neurodevelopmental condition (Panpalli Ates & Yilmaz Can, 2020; Zhou et al., 2021). Emerging research suggests a complex interplay between genetic susceptibility and environmental exposures, contributing to metabolic and immune anomalies across multiple organ systems, including the brain. These factors may influence cognitive function later in life. Among them, gastrointestinal (GI) health is gaining attention, with studies exploring how antibiotic exposure, hospitalization history, and distinct gut microbiota profiles may relate to autism, though the precise nature of these associations remains unclear.

Studies increasingly highlight a high prevalence of GI issues in autistic adults (Dargenio et al., 2023; Holingue et al., 2018). The gut-brain axis—a bidirectional communication system between the central nervous system and the GI tract—plays a critical role in both neurological and psychiatric conditions, including autism (Appleton, 2018). Persistent GI symptoms in autistic individuals suggest a potential link between gut microbiota, dietary habits, and aging-related changes.

Nutritional patterns among autistic individuals further complicate this relationship. Eating disorders and feeding difficulties are common, yet they differ in etiology and should be clearly distinguished in clinical assessments to guide effective interventions (Bertelli et al., 2025). Eating disorders, such as anorexia, bulimia, and avoidant restrictive food intake disorder (ARFID), often involve body image concerns and fear of weight gain. In contrast, autism-related feeding challenges stem from extreme food selectivity and sensory aversions, leading to a preference for starches and snack foods while rejecting fruits and vegetables (Bayoumi, 2025). However, the link between autism and both feeding and eating disorders remains poorly understood, limiting progress in clinical care (Adams et al., 2024). These dietary behaviors can profoundly affect nutrient intake, gut microbiome diversity, and overall gastrointestinal health, with broader implications for systemic well-being.

Comprehensive reviews suggest that interventions like probiotics may alleviate both GI and behavioral symptoms in autism (Zhang et al., 2024). However, large-scale, controlled studies are needed to confirm their mechanisms and long-term effects (Lewandowska-Pietruszka et al., 2023). The gut-brain connection is highly complex, influenced by microbial diversity, external environmental factors, and individual physiological differences. Understanding this interplay opens promising avenues for therapeutic development, including targeted treatments for GI disorders, cognitive function enhancement, and potentially delaying neurodegeneration. Further research on autistic adults may provide critical insights into the relationship between GI health and dementia risk, offering opportunities for early intervention.

### Recognition and Assessment

Diagnosing autism in adults is challenging and complex. Since its original descriptions by Ssucharewa (1926), Kanner (1943), and Asperger (1944/1991), the understanding of autism has evolved, and many individuals initially diagnosed as children are now reaching older age. While some symptoms may diminish over time, autistic adults often continue to face challenges in social interaction, communication, repetitive behaviors, sensory processing, and executive function—difficulties that can be further intensified by the aging process.

Two primary diagnostic criteria, the DSM-5 (American Psychiatric Association, 2022) and the ICD-11 (WHO, 2019), guide professionals in evaluating autism symptoms and their impact. Approaches to diagnosing adults vary; some guidelines recommend multidisciplinary assessments, while others suggest evaluation by a single experienced professional (AutismSA, 2023; CDC, 2022; Durant, 2013; National Institute for Health Care and Excellence, 2021; Royal College of Psychiatrists, 2020; Whitehouse et al., 2018). A comprehensive approach is crucial for adult diagnosis, incorporating developmental, psychiatric, and medical history along with core clinical features. Currently, no definitive biomarker for autism has been identified, reflecting the complexity and variability of its symptoms, and biomarkers validated in children have not yet been adapted for adults (Frye et al., 2019). However, ongoing biomarker research in Alzheimer’s disease and other dementias presents promising opportunities for cross-diagnostic breakthroughs that could be applied to autism.

### Characteristics and Variations

Autism assessments reveal notable differences between sexes, with the condition less frequently diagnosed in females. This disparity may stem from genetic and hormonal factors, as well as differences in symptom presentation. Women often exhibit social challenges that may appear more subtle as compared to men and are frequently masked or camouflaged, which can obscure autistic traits and delay diagnosis (Rynkiewicz et al., 2016). These challenges may include difficulties with friendships, sensitivity to textures, social anxiety, eating disorders, and mood disorders (Hull et al., 2017). While women may present with different autism traits to men, the impact of symptoms should not be underestimated.

The commonly cited 4:1 male-to-female ratio is influenced by cognitive levels, with males overrepresented among low support need individuals. Additionally, the emergence of autism-like symptoms later in life has been linked to degenerative conditions like young- or early-onset dementia. While dementia rates are higher in women, this is attributed to longer life expectancy (Beam et al., 2018) and to greater vulnerability for cognitive decline (Klein et al., 2013). Further research into the intersection of sex, autism, and late-life dementia could provide insights into the shared neuroanatomical mechanisms of these conditions.

### Co-occurring Autism and Other Neurodevelopmental Disorders

When examining neurodevelopmental disorders that co-occur with autism, it becomes clear that individuals with genetic or genomic conditions—such as attention-deficit/hyperactivity disorder (ADHD), fragile X syndrome, tuberous sclerosis complex, and Down and Rett syndromes—often exhibit autistic traits (CDC, 2022). These overlaps suggest shared genetic mechanisms underlying multiple neurodevelopmental conditions. Syndromic autism, which occurs in the context of specific genetic conditions like fragile X syndrome or Rett syndrome, provides important insights into the complex genetic etiology of autism. In these cases, autism arises from single-gene mutations, triplet repeat expansions, or chromosomal abnormalities, often accompanied by ID. Given that ID is present in approximately 40% of autistic individuals (CDC, 2009), autism in these contexts should be understood as part of the broader syndrome rather than as an independent comorbidity.

Additionally, epilepsy is more prevalent in individuals with both autism and ID, with seizure rates reaching up to 30% in autistic individuals (Rosen, 2020). Late-onset epilepsy has been identified as a key indicator of Alzheimer’s-related dementia in adults with Down syndrome, raising questions about its potential role in neurodegeneration. While studies have noted the overlap between epilepsy, autism, and ID (Lee et al., 2015), the direct link between epilepsy and dementia in autistic individuals remains unclear, emphasizing the need for further research.

The relationship between DS and autism shows varying prevalence rates, with a co-occurrence of about 2% in the U.S. population (Baio et al., 2018) and between 8 and 37% among children in the United Kingdom (Moss et al., 2013; Warner et al., 2014). Individuals with DS face a significantly higher risk of developing dementia, due to the buildup of amyloid-beta protein, a key feature of Alzheimer’s disease (Antonarakis et al., 2020). This overlap may stem from shared genetic factors and specific neuropathological changes, adding complexity to the understanding of co-occurring conditions. However, the implications of these findings for the risk of dementia in autistic individuals remain uncertain and warrant further exploration.

### Health and Related Factors

Syndromic autism, associated with genetic conditions such as fragile X syndrome and tuberous sclerosis complex, presents more complex health and medical challenges than non-syndromic autism. Individuals with syndromic autism often experience comorbidities such as epilepsy, metabolic disorders, and increased neurodegenerative risks, all of which contribute to cognitive decline and mobility issues. Syndromic autism can be categorized into two groups: (1) autism occurring within a clinically recognized syndrome, diagnosed through targeted genetic testing, and (2) autism associated with molecularly defined syndromes, identified via genome-wide sequencing (Fernandez & Scherer, 2017).

Research highlights a broad range of comorbidities in autistic adults, including seizures, gastrointestinal issues, psychiatric disorders, infections, immune dysfunction, hearing/sensitivity impairments and auditory processing disorders impairments (Brondino et al., 2019; Cawthorpe, 2017; McKenna et al., 2024; Miot et al., 2019; Robinson-Agramonte et al., 2022; Vohra et al., 2017; Wise et al., 2017). These conditions, influenced by genetic and familial factors, may contribute to progressive health challenges and an increased risk of dementia in later life. Given these complexities, there is a critical need for targeted healthcare strategies and preventive interventions to support aging autistic individuals and reduce neurodegenerative risks (Hamdan & Bennett, 2024). Examining syndromic autism in the context of aging provides essential insights into tailored healthcare strategies, long-term support needs, and the intersection of neurodevelopmental and neurodegenerative conditions.

## Factors Related to Autism and Dementia

Dementia, characterized by cognitive and functional decline, is more common among individuals with ID, especially those with DS (Lautarescu et al., 2017; National Institute on Aging, 2023). Among autistic adults, the risk of dementia varies based on the presence of co-occurring ID or DS. Recent studies indicate that autistic adults have a 2.6 times higher prevalence of young- or early-onset dementia compared to the general population (Vivanti et al., 2021). However, other research suggests that autistic individuals diagnosed in adulthood without ID may not face an increased risk of accelerated cognitive decline (Torenvliet et al., 2023). When cognitive decline does occur, males with autism seem to be at a higher risk for dementia than females.

Some studies suggest that autistic adults may have some protection against age-related cognitive decline (Oberman & Pascual-Leone, 2014), while others highlight potential links between autism and dementia (Rhodus et al., 2020a). Indicators of dementia in autistic adults can include a decline in frontotemporal functioning, increased severity of behavioral and psychological symptoms, more pronounced stereotypical behaviors, and heightened compulsivity (Rhodus et al., 2023). Understanding this relationship is complex due to overlapping behavioral and cognitive symptoms, communication challenges, limited verbal expression, and atypical presentations of dementia. Further research is essential to clarify the connection between autism and dementia in older adults.

### Risk for Dementia

Research shows that autistic adults under the age of 65 are approximately 2.6 times more likely to be diagnosed with younger-onset dementia compared to the general population; however, these data do not establish a direct causal link between autism and Alzheimer’s disease (Vivanti et al., 2021). Instead, these findings may reflect various forms of dementia or potential misdiagnoses. The relationship between autism and specific types of dementia remains complex and unexplored. Additionally, data on the prevalence of Alzheimer’s disease among older autistic adults are scarce, and current evidence suggests that they may not experience higher rates of Alzheimer’s dementia in later life, however, if dementia occurs, it may be attributed to other factors (e.g. social exposome; Kind et al., 2025). Overall, the interplay between autism and dementia in older adults necessitates further investigation to clarify potential connections and risks. Furthermore, the risk of “diagnostic overshadowing” in the diagnostic process may hinder accurate interpretation of these conditions.

Reviews indicate that the average age at death for autistic adults is like that of the general population, except for those with significant comorbidities. Notable mortality differences exist between individuals who are relatively independent and those who require substantial support. Mortality rates are also particularly higher among individuals with both autism and intellectual disabilities (Hirvikoski et al., 2016).

Sex differences are also evident, with autistic females having a higher mean age at death than males, mirroring trends in the general population (Lunsky et al., 2022). Autistic individuals are less likely than their non-autistic counterparts to have Alzheimer’s disease or dementia listed as a cause of death (Catalá-López et al., 2022). However, males with autism are more likely than females to have dementia noted on their death certificates (Barnard-Brak et al., 2019). Differences in causes of death by sex reveal that women with autism are more prone to deaths associated with endocrine disorders and birth defects, while men face higher risks from nervous and circulatory system disorders (Hirvikoski et al., 2016). Additionally, epilepsy is a common cause of death in autistic adults with high support need levels, whereas circulatory diseases are more prevalent among those with low support need levels. Overall, higher mortality rates in autistic individuals are often attributed to lifestyle and social factors rather than intrinsic genetic elements. These findings highlight the need for further research to better understand mortality factors, including the role of dementia, within the autistic population.

### Risk Factors

As research continues to identify risk factors affecting various populations, the influence of social determinants, individual behaviors, environmental factors, and biological systems on health outcomes has become increasingly clear (Livingston et al., 2024). Understanding the intersection of these determinants is essential for addressing health disparities, particularly among adults with disabilities. Government agencies are actively investigating these disparities (NIMHD, 2023).

Researchers adapting frameworks for mental health disparities highlight the importance of both biological factors—such as allostatic load and inflammatory responses—and sociocultural influences like stigma and bias. Allostatic load, which refers to the physiological response to chronic stress, is proposed as a mechanism contributing to physiological dysregulation and accelerated aging in autistic adults. This concept is like the ‘weathering hypothesis’ used to explain health disparities among certain ethnic, racial, or social minority groups (Geronimus, 1992; Johnson & Gomez, 2023). The interplay between biological and sociocultural factors offers a framework for understanding the health challenges faced by autistic adults, including accelerated aging driven by multiple stressors and immune system dysregulation. Additionally, disruptions in circadian rhythms and sleep, combined with the effects of allostatic load, complicate the understanding of dementia-related health risks specific to autism.

#### Intellectual Disability and Risk for Dementia

The relationship between ID and dementia in individuals with autism is complex and mostly unexplored. Research indicates a higher prevalence of cardiovascular risk factors among autistic individuals, particularly those with co-occurring ID. These factors include overweight, obesity, diabetes, and hyperlipidemia, all of which are linked to dementia in people with ID (Bishop-Fitzpatrick & Rubenstein, 2019; Cashin et al., 2018; Takenoshita et al., 2023).

Studies utilizing U.S. Medicaid data have found that autistic adults with ID have increased odds of neurological disorders, including dementia, compared to their counterparts without ID (Vivanti et al., 2025). However, the observed differences in physical and mental health conditions were not statistically significant, suggesting that comorbidities may play a more substantial role in the association with dementia than autism itself (Bishop-Fitzpatrick & Rubenstein, 2019).

While the connection between autism and dementia is less clear in the absence of concomitant ID, research points to shared genetic and neurobiological factors, particularly with forms such as frontotemporal dementia (Midorikawa & Kawamura, 2012; Rhodus et al., 2020b). Some studies have suggested a potential link between autism and Parkinson’s disease (Mai et al., 2023). The recognition that dementia is a common feature of Parkinson’s disease further complicates the understanding of the interplay among autism, ID, and various forms of dementia. This underscores the need for further research to differentiate among these conditions and investigate their underlying mechanisms, including medication exposure and pathogenetic factors.

#### Down Syndrome and Risk for Dementia

Studies indicate that from eight to 37% of individuals with DS are also diagnosed with autism (Dimachkie Nunnally et al., 2021; Richards et al., 2015). Those persons with both DS and autism are more likely to experience epilepsy, gastroesophageal reflux, and constipation. Given that adults with DS commonly exhibit neuropathological changes associated with Alzheimer’s disease by age 40, it raises further concerns about the risk of dementia in individuals with both DS and autism (Rubenstein et al., 2020). However, dedicated research on dementia within this population remains scarce.

A population-level study indicated that autistic adults with co-occurring ID are 2.5 times more likely to be diagnosed with young- or early-onset dementia compared to the general population (Vivanti et al., 2021). However, since this study did not specifically analyze individuals with DS, some adults with ID who also had DS may not have been identified. Conversely, another study found that adults with mosaicism or mosaic Down syndrome are more likely to be diagnosed with autism than those without mosaicism (Rubenstein et al., 2024). While the coexistence of DS and autism is acknowledged, any increased risk of Alzheimer’s disease in adults with both conditions may be more closely associated with the presence of DS rather than autism itself. This attribution, however, still requires further evaluation.

#### Psychiatric Conditions and Risk for Dementia

Multiple studies have highlighted the intricate relationship between autism, cognitive health, and cognitive decline in adulthood (e.g. Hand et al., 2020; Vohra et al., 2017). Diagnosing autism in adults can be challenging due to comorbid psychiatric or neurological conditions that may obscure the manifestations of neurodevelopmental disorders. Adults with mental health disorders, including those with autism, are at an increased risk of dementia, which can develop earlier than in individuals without significant mental health disorders (Richmond-Rakerd et al., 2022). Psychiatric symptoms, such as self-injurious and disruptive behaviors, further contribute to this risk; rates of these disorders are nearly doubled in adults with both ID and autism compared to those with ID alone (Kats et al., 2013). Cross-sectional and longitudinal studies suggest that autistic adults without ID experience cognitive decline and challenges with executive functioning with age similar to non-autistic adults, indicating that the heightened dementia risk may be more pronounced among those with concomitant ID (Powell, Klinger, & Klinger, 2017; Torenvliet et al., 2023). Also, some aspects of memory loss and cognitive decline in autistic adults with co-occurring psychiatric conditions may be linked to the extended use of potent anticholinergic medications (McQuaid et al. (2024). Additionally, findings indicate that older adults with elevated autistic traits may experience poorer mental health in later life, with mental health difficulties persisting into old age without improvement. These findings highlight the need for further research to clarify the complex relationships among autism, cognitive decline, and potential protective factors such as brain plasticity.

#### Behavioral and Psychological Symptoms and Risk for Dementia

Autistic individuals often experience increased social isolation and mental health challenges, frequently meeting the criteria for comorbid mood and anxiety disorders that may also resemble behavioral and psychological symptoms of dementia (BPSD) (Cheney et al., 2023). However, the impact of autism on the manifestation of BPSDs is not well understood. Some studies have noted similarities between many BPSDs, and symptoms associated with childhood autism spectrum disorder, including anxiety, depression, and communication deficits (Rhodus et al., 2023). Yet, the late-life onset of behaviors typical of autism has not been thoroughly examined.

In individuals with DS, BPSD symptoms—such as increased anxiety, sleep disturbances, apathy, and depression—may indicate the onset of Alzheimer’s disease or progression to dementia (Dekker et al., 2018). The co-occurrence of DS may heighten the risk of Alzheimer’s disease in autistic adults, complicating the overall picture. For autistic adults with DS, heightened verbal or physical aggression may signal mild cognitive impairment or early dementia related to Alzheimer’s disease. It remains unclear whether and to what extent BPSDs manifest in other forms of dementia among autistic adults. Recognizing BPSDs is critical for timely intervention and support. This area warrants further in-depth research to better understand and address the complex interplay of these factors.

## Dementia Variants and Autism

The literature indicates a significant association—though not necessarily a causal relationship—between autism and frontotemporal dementia (FTD). FTD encompasses a spectrum of neurodegenerative disorders, primarily affecting individuals under 60 years of age, and is characterized by a notable hereditary component. The onset of FTD can vary widely within families, with some experiencing a decade-long range of intrafamilial variability. Familial heritability is associated with an increased risk of psychiatric disorders, including schizophrenia and autism (Greaves & Rohrer, 2019). There is evidence of overlap between behavioral variant FTD (bvFTD) and autism, with some older adults displaying autism-like behaviors that resemble symptoms of bvFTD (Hodges, 2023). Additionally, late-onset autism symptoms, such as seizures, have been observed in individuals with dementia, particularly Alzheimer’s disease (Rhodus et al., 2020b). Neuropathological evaluations have shown increased tau and neurofibrillary pathology in the frontal lobes of adults exhibiting autism-like behaviors in late-onset dementia. Diagnosing bvFTD remains challenging due to symptom overlap with psychiatric disorders. Pursuit of research utilizing biomarkers could enhance diagnostic clarity (Ducharme et al., 2020; Greaves & Rohrer, 2019).

Emerging research suggests a link between neurodevelopmental conditions such as autism and ADHD and neurodegenerative disorders like Parkinsonism and dementia (Midorikawa & Kawamura, 2012). Activation of microglia is conducive to neurotoxicity as a principal immunopathological mechanism taking place in both autism and Parkinson disease (Robinson-Agramonte et al., 2022). While the relationship between autism and dementia remains complex and inconclusive, studies indicate a more consistent association between autism and Parkinsonian features (Geurts et al., 2022; Hand et al., 2020; Starkstein et al., 2015). However, most research has focused on the motor symptoms of Parkinsonism in autistic adults, leaving a critical gap in understanding how these movement-related impairments may interact with cognitive function and dementia risk. The mechanisms underlying this connection remain unclear, but early neurodevelopmental differences, combined with factors such as reduced physical activity and social isolation, may contribute to cognitive decline in autistic individuals. Additionally, behavioral and cognitive characteristics of autism—such as repetitive behaviors and hyper focused interests—may influence neurodegenerative processes, potentially increasing dementia risk. Limited social engagement, which is common among autistic individuals, could also reduce cognitive resilience, further compounding this risk. Given the higher prevalence of Parkinson’s dementia and Parkinsonian syndromes in autistic adults (Mai et al., 2023), further research is needed to clarify the cognitive consequences of co-occurring Parkinsonism and autism. A deeper understanding of how neurodegeneration manifests in autistic individuals could inform targeted interventions addressing both motor and cognitive symptoms, ultimately helping to mitigate dementia risk and improve long-term outcomes.

## Commentary

The data reviewed for this report did not indicate a significantly elevated risk of Alzheimer’s disease among autistic individuals. However, some data suggest a higher prevalence of other types of dementia, such as behavioral variant frontotemporal dementia.

Our findings highlight the complex relationship between autism and dementia, which encompasses various factors, including behavioral manifestations, genetic susceptibility, shared neurobiological processes, and environmental influences. The interplay among ID, autism, and dementia involves a combination of genetic, neurobiological, and environmental factors. Addressing these multifaceted challenges necessitates a comprehensive approach that integrates assessment, intervention, and support tailored to each individual’s unique needs and circumstances. Such an approach is vital for developing targeted prevention and intervention strategies, identifying early biomarkers, and exploring potential disease-modifying treatments for any existing dementia.

We propose that a holistic, person-centered approach is essential for effectively navigating the complexities of aging with neurodevelopmental conditions. Recognizing and addressing the unique needs and challenges faced by individuals with autism, with and without ID, and dementia is crucial for providing optimal care and support. Continued research and collaboration among healthcare professionals, researchers, and advocacy groups are vital for enhancing our understanding and improving the quality of life for this population (Klein & Klinger, 2024).

The A/DWG review highlighted that previous attempts to define the intersection of dementia and autism have not produced clear and conclusive results. While we observed connections between certain life factors, bio-neurological processes, and dementia risk in autistic adults, a definitive relationship between the onset of neurodegenerative disorders and behavioral expressions of dementia remains elusive. Although the literature supports associations between adverse social determinants of health and clinical expressions of dementia, a clear trajectory for autistic adults affected by dementia has yet to be established.

Further research is needed to elucidate the intricate relationships among genetic, neurobiological, and environmental factors contributing to the heightened risk of neurodegenerative disorders or dementia in autistic individuals, particularly among those with co-occurring ID, especially as their average lifespan increases. Findings from such studies will be crucial for developing targeted prevention, mitigation, and intervention strategies.

The Summit emphasized the importance of brain health alongside evidence-based practices that strengthen social skills, support healthy lifestyle choices, and provide tailored living assistance. These practices aim to empower autistic individuals, respecting their consent and preferences, while reducing exposure to unsafe environments and risk-enhancing behaviors. The goal is to foster adherence to lifestyle and habits that promote overall well-being, including both mental and physical health, thereby supporting lifelong brain health and resilience.

Addressing these emerging life practices and research issues will require enhanced international collaboration among universities, research institutes, civil society organizations, government agencies, and family advocacy groups as well as the allocation of resources for studying the intersectionality of aging, autism, and related neuropathologies. Additionally, it is important to continue convening study groups to explore and integrate new findings.

## Conclusion

Current research indicates that there is no substantial evidence linking autism with a *significantly increased risk* of specific types of dementia. As autistic individuals age, they may receive dementia diagnoses at rates similar to those in the general population, but we found no clear genetic predispositions to particular brain diseases – although the absence of a predisposition does not negate there potentially being one. However, adults with co-occurring conditions, such as Down syndrome and other intellectual disabilities, may face a higher risk and experience elevated rates of clinical dementia in later life. The report from the 2nd International Summit on Intellectual Disability and Dementia emphasized the impact of structural and social determinants, adverse life experiences, and stressors on cognitive health in later life. While the research remains preliminary, it is not yet clear whether these factors lead to earlier or more severe dementia outcomes for autistic adults compared to the general population. As preventative measures, the Summit’s report advocates for evidence-based practices that enhance social skills, promote healthy lifestyles, and provide supportive living environments. These strategies aim to sustain brain health and overall wellness among autistic adults, potentially minimizing exposure to unsafe conditions and encouraging behaviors that support positive mental and physical health.

## Footnotes

1 Autism, Dementia, and Diagnostics: A Consensus Report from the 2nd International Summit on Intellectual Disabilities and Dementia, in submission.

2 Autism, Dementia, and Post-Diagnostics Supports: A Consensus Report from the 2nd International Summit on Intellectual Disabilities and Dementia, in development.
